# Integrative omics approaches for biosynthetic pathway discovery in plants

**DOI:** 10.1039/d2np00032f

**Published:** 2022-08-23

**Authors:** Kumar Saurabh Singh, Justin J. J. van der Hooft, Saskia C. M. van Wees, Marnix H. Medema

**Affiliations:** Bioinformatics Group, Wageningen University Wageningen The Netherlands marnix.medema@wur.nl; Plant-Microbe Interactions, Institute of Environmental Biology, Utrecht University The Netherlands s.vanwees@uu.nl; Department of Biochemistry, University of Johannesburg Auckland Park Johannesburg 2006 South Africa

## Abstract

Covering: up to 2022

With the emergence of large amounts of omics data, computational approaches for the identification of plant natural product biosynthetic pathways and their genetic regulation have become increasingly important. While genomes provide clues regarding functional associations between genes based on gene clustering, metabolome mining provides a foundational technology to chart natural product structural diversity in plants, and transcriptomics has been successfully used to identify new members of their biosynthetic pathways based on coexpression. Thus far, most approaches utilizing transcriptomics and metabolomics have been targeted towards specific pathways and use one type of omics data at a time. Recent technological advances now provide new opportunities for integration of multiple omics types and untargeted pathway discovery. Here, we review advances in plant biosynthetic pathway discovery using genomics, transcriptomics, and metabolomics, as well as recent efforts towards omics integration. We highlight how transcriptomics and metabolomics provide complementary information to link genes to metabolites, by associating temporal and spatial gene expression levels with metabolite abundance levels across samples, and by matching mass-spectral features to enzyme families. Furthermore, we suggest that elucidation of gene regulatory networks using time-series data may prove useful for efforts to unwire the complexities of biosynthetic pathway components based on regulatory interactions and events.

## Abstract 2

This review discusses genomics-, transcriptomics- and metabolomics-based plant biosynthetic pathway discovery, and highlights the potential for integrative omics approaches.

## Graphical abstract

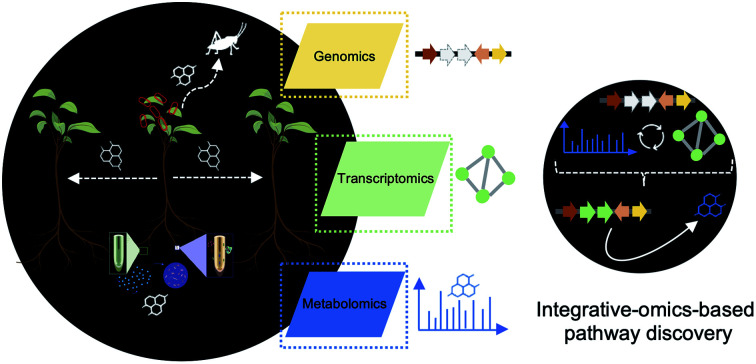

## 1. Background

**Fig. 1:**
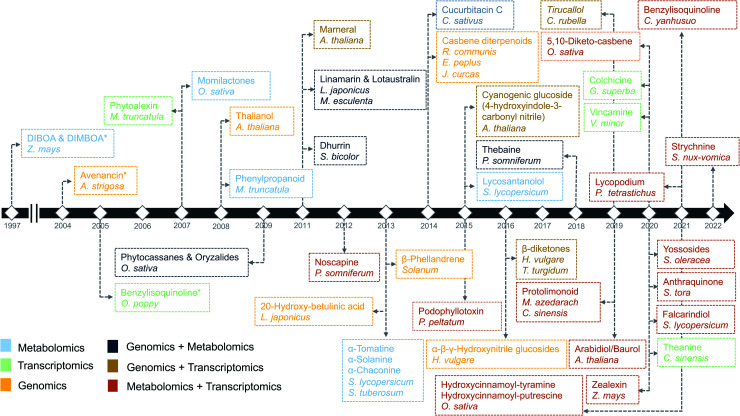
Timeline of the identification of biosynthetic pathways in plants. The names of secondary metabolites and the associated species/genus are color coded based on the omics technology used in the identification process.^7,9,14,21–23,97,106,112,113,134,144,146–172^ An asterisk means the initial discovery of biosynthetic genes using genetics and/or biochemical-based approaches.

Plants are sessile organisms and therefore, unlike animals, are unable to circumvent adverse environmental conditions. However, with constantly varying pressures across evolutionary time scales, they have learned to combat stress by producing a myriad of specialized metabolites, also known as natural products (NP). These specialized metabolites have been recognized to serve important ecological and physiological roles such as plant growth modulation,^1^ conferring protection against biotic stress and mediating interactions with other plants, insects, and microbes.^2^ In the past decades, the technological improvements and cost reductions in generating high-throughput omics datasets from plants, together with the development of computational genome mining tools, have led to rapid advancements in the discovery of biosynthetic pathways responsible for specialized metabolites synthesis.^3^ More than 30 biosynthetic gene clusters (BGCs) and many non-clustered biosynthetic pathways^4^ have been fully characterized so far in the plant kingdom^2^ (Fig. 1). However, despite these advances, the genetic complexity and functional diversity of plant biosynthetic pathways still pose a large challenge to the scientific community.^5^ Although single-omics-based studies, utilizing genomics, transcriptomics, or metabolomics, have facilitated the characterization of selected biosynthetic pathways and their metabolic products, systematic approaches to rapidly identify partial or complete pathways in an untargeted manner have been lacking. Integrative omics approaches have recently emerged and proven useful for the elucidation of plant metabolic pathways.^6^ In this review, we report the contributions of single-omics technologies and emphasize the importance of integrative omics as a comprehensive approach to plant biosynthetic pathway discovery. Specifically, we highlight how new technologies to unravel gene regulation could augment current approaches. Finally, we discuss the prospects and challenges of multi-omics data integration.

## 2. Genomics

In the past decade, plant specialized metabolism research has benefitted immensely from the availability of increasing numbers of genomes and large amounts of functional genomic data. The first discovery of “gene clusters” in plants dates back to 1997, when genes involved in the biosynthesis of 2,4-dihydroxy-7-methoxy-1,4-benzoxazin-3-one (DIMBOA) were found to be clustered on a single chromosome in maize^7^ (Fig. 1). Though the presence of BGCs in plants was originally unexpected, this discovery has given rise to the perception that, similar to the situation in bacteria and fungi, plant genes involved in biosynthetic pathways tend to be co-localized.^8^ Several pathways were also discovered partially based on the concept of gene clustering. For example, bioinformatic interrogation of the genomes of *Solanaceae* led to the identification of genes encoding the biosynthetic enzymes for the production of steroidal glycoalkaloids.^9^ Furthermore, various common gene families involved in metabolite transformations, such as cytochrome P450s and terpene synthases, are frequently found in gene clusters.^10^ It is important to note here that gene clusters in plants have previously been defined as ‘genomic loci encoding genes for a minimum of three different types of biosynthetic reactions (*i.e.*, genes encoding functionally different (sub)classes of enzymes)’,^10^ to distinguish them from tandem arrays. This definition is also used by plant BGC identification tools such as plantiSMASH.^11^

Despite technological advancements, however, until now only 30–40 BGCs have been completely characterized in plants. This moderate progress in the discovery of biosynthetic pathways owes partially to the plant genome complexity and partially to the fact that clustering of biosynthetic genes, unlike in many bacteria and fungi, is not ubiquitous in the plant kingdom. Even the identification of clustered biosynthetic genes does not guarantee the identification of a biosynthetic pathway, because plant genomes contain groups of duplicated genes in tandem arrays that often do not encode entire pathways. Interestingly, some tandem arrays, *e.g.*, a set of tandem-duplicated cytochrome P450 (CYP) genes involved in DIMBOA biosynthesis in maize^7^ or the tandem array of methyltransferase genes involved in caffeine biosynthesis in *Coffea canaphora*,^12^ do encode subsequent steps in the pathway. Another complication in pathway discovery is that, even if the majority of genes of a biosynthetic pathway are present in a gene cluster, some of the pathway genes may be located at different loci. For example, GAME7 (glycoalkaloid metabolism 7), a gene from the CYP72 subfamily, catalyzes the first step in the biosynthesis of steroidal glycoalkaloids in tomatoes and is located on the same chromosome, but it is separated by approximately 8 Mb from the other pathway genes.^9^ In monoterpene indole alkaloid biosynthesis, various sets of genes involved in different parts of the pathways have been found to be located in different gene clusters.^13^ For many other biosynthetic pathways, *e.g.*, for glucosinolates, flavonoids, and anthocyanins, genes are (mostly) scattered throughout the genome.^14^

Advancements in sequencing technologies have benefitted the biosynthetic pathway discovery process. To date, around 300 complete chromosome-scale genome assemblies have been generated.^15^ The generation of such assemblies for plants remains a challenging task and is therefore lagging behind the generation of genome-scale sequencing data. This can be attributed to complexities within plant genomes like variation in genome size, highly variable percentage of transposable elements and other repetitive DNA content ranging from 3% to 85%. Repeated occurrence of whole genome duplications (WGDs) or ploidy in the genome makes the assembly highly challenging. Due to variable ploidies, total count of the genes within a genome also appears to be variable with in plant families, with an abundance of pseudogenes. In addition to handling genomic complexities, completion of a genome requires annotation efforts to accurately describe gene structure (in particular intron–exon boundaries) as well as order and orientation. Artifacts in the annotation may lead to incorrect inference of gene family and its function. Such errors may also get propagated to new assemblies and public repositories as more genomes are assembled. Hence, quality of assembly (completeness and contiguity) and annotation have major impacts on the prediction of biosynthetic genes and their regulation and function in plants. An eminent example of the usage of high-quality genome assembly for the prediction of biosynthetic pathway genes is the characterization of the last stages of the avenacin pathway. The discovery of the first gene in the avenacin pathway, beta-amyrin synthase-encoding *AsbAS1*, was based on gene cloning and predicting the enzyme class using enzymatic assays.^16^ Candidate genes required for the avenacin biosynthesis, were later prioritized based on linkage-mapping, where the *AsbAS1* gene was positioned on the genetic map using genetic markers, and subsequently, other linked pathway genes were identified using a combination of genetics, using avenacin-deficient mutants, and physical proximity (as determined by BAC libraries).^16^ More recently, the remaining two steps (characterization of *CYP94D65* and *CYP72A476* genes) in the avenacin pathway have been characterized by assembling a high-quality oat genome using the latest sequencing approaches.^17^ Importantly, in the past decades, at least nine biosynthetic pathways have been characterized using genomics-based approaches (Fig. 1).

The recent development of computational approaches has also expedited the discovery of biosynthetic pathways. For example, plantiSMASH^11^ allows the identification of biosynthetic genes using a comprehensive library of plant-specific profile Hidden Markov Models (pHMMs) for key specialized metabolic enzyme families, in combination with CD-HIT clustering of the predicted protein sequences to distinguish gene clusters encoding diverse enzymes from tandem arrays. Schläpfer *et al.* used a sliding window approach to mine BGCs in plant genomes,^8^ and Töpfer *et al.* developed PhytoClust^18^ to explore plant genomes for BGCs using a system similar to that of plantiSMASH. With the development and uptake of such algorithms for the identification of biosynthetic genes in plant genomes, it is evident that identifying a group of co-localized genes is a viable strategy for specialized metabolic pathway discovery. However, genomics alone is not sufficient to confidently and precisely identify plant specialized metabolic pathways because co-localization of genes neither guarantees coexpression nor co-involvement in the same pathway. To overcome these challenges, recent computational tools allow the use of transcriptomics data to measure coexpression among biosynthetic genes both within and across genomic loci.

**Fig. 2:**
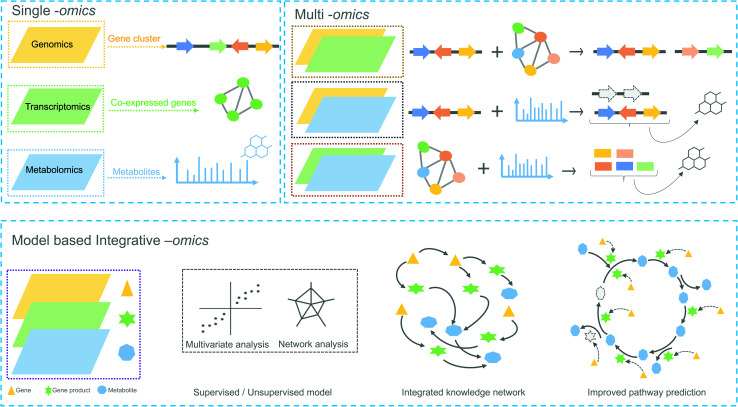
Overview of omics experiment designs to elucidate secondary metabolic pathways. Top: single and combination of omics design result in mapping individual genes, proteins or metabolites to a set of pathway components. Bottom: an integrative-omics approach combines knowledge from different layers of a biological system and can be used for generating an integrated knowledge network (IKN). The IKN enables the identification of hidden interactions between genomic features and unravels the regulation of genes across time points and different conditions. Integrative omics likely better predicts the different components of a biosynthetic pathway than single- or multi-omics. Dashed lines in the genetic architecture and pathway indicate missing/unknown components.

In the following sections, we highlight how complementary omics approaches, for instance, transcriptomics and metabolomics, and how their integrative analysis can add key information to locate and identify novel plant biosynthetic genes and metabolic pathways (Fig. 2).

## 3. Transcriptomics

Understanding the pathways involved in plant specialized metabolism and their regulation requires the investigation of genes encoding enzymes, transcription factors, and transmembrane transporters. RNA (ribonucleic acid) sequencing (RNA-seq), currently the most widely used transcriptomic technology,^19^ has lately been routinely used to capture genome-wide expression patterns of genes.

### 3.1 Transcriptomics in plant specialized metabolism

Transcriptomics has guided pathway discovery, as both clustered and distal genes involved in biosynthetic pathways share similar expression patterns across conditions and time points^20^ (Fig. 1). For example, noscapine biosynthetic genes were characterized in 2012 using pyrosequencing from ESTs (Expressed Sequence Tags) libraries based on the principle of coexpression.^21^ Later, genes involved in the biosynthesis of podophyllotoxin in mayapple and 4-hydroxyindole-3-carbonyl nitrile (4-OH-ICN) in *Arabidopsis* were successfully elucidated by mining publicly available transcriptomic datasets.^22,23^

### 3.2 Coexpression analysis

**Fig. 3:**
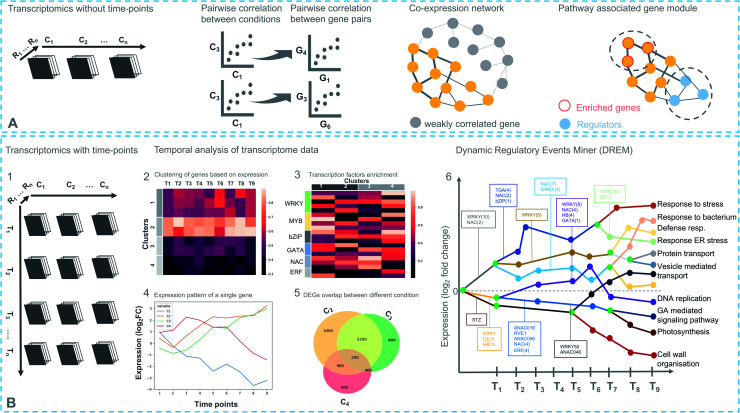
Different strategies for transcriptomics-based analysis. (A) Experimental design with different conditions (C), without time-points (top). Coexpression networks constitute a useful method to identify genes with similar expression patterns, which may belong to a biosynthetic pathway. (B) (1) Experimental design with different conditions (C) and time points (*T*) (bottom). (2) Differentially expressed genes in response to a treatment are partitioned into clusters based on their coexpression. Each row represents a single gene and its expression at different time points. (3) Enriched *cis*-regulatory motif in gene coexpression modules. (4) Expression pattern of a single regulator at different time points under different conditions. (5) Comparing degree of overlap between different treatments. (6) Simplified DREM model annotated with TFs. Each path corresponds to a set of coexpressed genes. Green nodes are the bifurcation points where coexpressed genes diverge in expression.

Coexpression analysis using RNA-seq data has been successfully applied in the discovery of pathways producing multiple classes of plant-based secondary metabolites like tri-, di-, or mono-terpenes, glycoalkaloids, glucosides, fatty acids, benzoxazinoids, acyl sugars, *etc.*^2^ (Fig. 1). This also facilitates the assignment of gene functions, using the guilt-by-association principle, to novel biosynthetic genes.^24^ Hansen *et al.* applied comparative transcriptome analysis using coexpression networks to elucidate the function of genes involved in cellulose biosynthesis. They observed conservation in biological pathways across different plant species and used this to transfer annotations from model to non-model plant species.^25^ To measure coexpression among any two genes, a variety of statistical correlation-based approaches are commonly used, for instance, Pearson correlation or Spearman's rank correlation. In the most often used—targeted—approaches, prior knowledge on function of a bait gene is required, to propagate annotations to unknown genes. Obtaining such knowledge is laborious due to the massive gene count of plant genomes. To bypass this obstacle, network-based approaches have been routinely adopted to decipher coexpression patterns (Fig. 3A). Here, individual genes are represented as nodes connected by the edges that show a strong expression correlation with other genes. Crucially, determining the edge weight threshold is a major stumbling block in identifying biologically significant correlations.^26^ Despite this limitation, Mao *et al.* generated a coexpression network using 1094 microarray datasets of *Arabidopsis* and reported functional categorization of genes by grouping them into modules of similar function or regulation.^27^ Cutoff scores used to generate coexpression networks are often debatable and arbitrary.^28^ In the process of determining a biologically meaningful correlation coefficient for a coexpression network associated with drought responsiveness in rice, Zhang *et al.* compared the actual number of edges in the coexpression network with all possible edges in the control network at different *r* cutoff values. They observed that as the *r* value moves from 0 to 1, the network density (defined as the ratio of the actual number of edges to the total number of edges) initially dropped to a minimum value and then increased drastically after *r* = 0.7. This increase in the network density was due to the presence of high *r* values links that are connected to a decreasing number of nodes.^28^ This indicated that biologically meaningful correlations are expected to be found at high *r* values. To reduce the size of the correlation network, different criteria can be applied like filtering for differentially expressed genes, or for genes with protein domains of interest. Alternatively, a cross-species comparison of the network can be performed to assess which pairs of genes show evolutionarily conserved coexpression patterns.

Comparative coexpression analysis has also been useful to identify homologous genes across species belonging to similar biosynthetic pathways. A notable example is the elucidation of the α-solanine/chaconine biosynthesis pathways in *Solanum tuberosum* and *Solanum lycopersicum*.^9^ While such coexpressed modules are mostly found to be conserved in species belonging to the same family, it is difficult to find conservation in evolutionarily distant species. Interestingly, finding the right combinations of multiple conditions or treatments can be used to extract biologically relevant modules from a regular coexpression network, combined with differential gene expression (DGE) analysis.^29^ Such methods, also known as differential coexpression methods, uncover complex relationships between genes by identifying changes in coexpression patterns across different conditions.^30^ Differential coexpression also aims to identify the variation of gene regulation across conditions through shared signaling pathways or transcription factors (TFs). Interestingly, a software package known as *dcanr*^31^ has been developed that encapsulates multiple methods to perform differential coexpression analysis on transcriptomics data.

### 3.3 Time-based omics

Coexpression of course is a symptom of a deeper layer of interactions, taking place at the level of transcriptional regulation. Despite its usefulness in gene function inference, some known limitations impede its utility in biological pathway discovery. For example, regulation of genes that do share similar functionality may be coordinated at the post-transcriptional level. It is also possible that genes that appear coexpressed do so because of the parameters used in the analysis. Uygun *et al.* reported that only 41% of Gene Ontology-Biological Process (GO-BP) terms have higher expression coherence (EC), *i.e.*, overall similarity of the expression profiles of genes involved, than expected by chance.^32^ Such differential regulation varies depending on the biological conditions, which means transcriptional regulation possesses the ability to re-wire in response to environmental triggers. Transcriptional responses in plants change over time and are achieved by a combined action of multiple TFs (including feed-forward and feed-back loops that can allow target genes to fluctuate in their expression patterns) that work synergistically to cause a genome-wide transcriptional cascade.^33^ A time-based study design can systematically capture gene expression fluctuations at different time points across multiple conditions. This increases the effectiveness of differential coexpression analyses. It also enables the reconstruction and modeling of gene regulatory networks (GRNs) by specifying TFs that temporally regulate gene expression.

Time-based studies have enhanced our understanding of the dynamic regulation by phytohormones, for example, ethylene,^34^ jasmonic acid,^35,36^ salicylic acid,^37^ and abscisic acid,^38^ which are key players in plant growth and defense. There are multiple other notable examples of studies that have generated key biological insights in different plant processes and responses, based on dynamic GRN inference.^39,40^ Time-series transcriptomic analyses have also been successfully applied to decipher biosynthetic pathways, for instance, theanine (thea) biosynthesis.^41^ Like all plant specialized metabolic pathways, thea biosynthesis involves a complex GRN with multiple TFs, structural and functional genes. The time-course experimental design was useful in this case, as it facilitated the determination of temporal effects of NAC (NAM/no apical meristem, Petunia, ATAF1–2/*Arabidopsis thaliana* activating factor, and CUC2/cup-shaped cotyledon, *Arabidopsis*) and bZIP (basic leucine zipper) TFs in the activation of thea biosynthesis.

A time-series experimental design can be represented as a three-dimensional matrix in which the *X*- and *Y*-axes correspond to samples from different conditions at different time points, respectively (Fig. 3B). Differential expression analysis follows the same approach described above for individual samples including pairwise comparisons of the time points. A recent report by Spies *et al.* has evaluated the performance of nine time-series-based differential expression analysis software on both simulated and biological data. The results were evaluated based on standard classification terms like true-positive, false-positive and false discovery rates *etc.*, with a stringent *p*-value cutoff of 0.01. The results based on the simulated data were further validated using a published biological dataset.^42^ Interestingly, the traditional pairwise comparison methods as implemented in EdgeR^43^ and DEseq2 (ref. 44) outperformed other time-based differential expression analysis methods on short time series (fewer than eight time points). On longer time series, the performance of splineTC^45^ and maSigPro^46^ was better than pairwise methods in terms of false-positive identification. Additionally, rmRNAseq^47^ was developed to accommodate correlation biases within the same experimental units in differential gene expression analysis involving repeated measures.

Pairwise-comparison-based methods cluster differentially expressed genes (DEGs) for individual conditions or jointly (co-clustering) by combining two or more different pairs of conditions and time points. In each instance, clustering is performed using a range of precision values to identify the most informative set of clusters, accounting for within-*versus* between-cluster variation. Later, individual clusters are annotated with GO annotations and TF families by performing enrichment and overrepresentation analysis from the Gene Ontology Resource and PlantTFDB,^48^ respectively. At this stage, TF DNA-binding motifs can be analyzed by using published position-specific weight matrices and experimentally defined TF-binding sites (TFBS) from *e.g.* JASPAR.^49^ Moreover, novel TF-target interactions can be inferred from ChIP-sequencing (ChIP-seq). However, the generation of gene-specific antibodies limits the throughput of this approach.^50^ DNA affinity purification sequencing (DAP-seq) can be used as an alternative approach in regions of accessible chromatin. To this end, O'Malley *et al.* defined the *Arabidopsis* cistrome (complete set of TFBS or *cis*-elements) by curating 529 TFs using DAP-seq data^51^ (https://www.neomorph.salk.edu/PlantCistromeDB).

Another way to analyze time-series data is by reconstructing dynamic GRNs using the Dynamic Regulatory Events Miner (DREM)^52^ method, which integrates time-series and static data using an Input-Output Hidden Markov Model (IOHMM). This method identifies so-called bifurcation points where a group of coexpressed genes (clusters) start to diverge. It then annotates the bifurcation points, using static/dynamic TFBS data, with TF(s) that control the split in the expression pattern. Although DREM is successful in reconstructing GRNs not only for plant species but other species as well, most current GRN models are based on TF-target interaction data at one or a limited number of time points, which limits the identification of (novel) regulatory TF(s) functioning in pathways.

### 3.4 Novel ways to construct a GRN by integrating dynamic and static omics data

GRNs can be built by using transcriptomics data in a correlation-based coexpression analysis. When based on one or a limited number of time points, this approach has two main caveats: first, these coexpression networks are nondirectional, and second, such an approach cannot discriminate between direct and indirect TF interactions (primary and secondary TF targets). To account for this limitation, coexpression networks are supplemented with time-series data. Such an approach can reveal the temporal order within a GRN and facilitates identifying directionality within the coexpression network.^33,53^ The activity of a TF precedes the expression of a target gene, and this delay could obscure the TF-target gene interactions in coexpression networks. Tools have been developed to account for such time lag and applied to correlation networks.^53^ To infer a causal relationship between TF and their target genes, machine learning approaches are being used, and multiple tools have been developed that integrate time-based transcriptomic and regulatory data into a machine learning model. Models developed for time-series data use the expression of predictor genes at one time point to model the expression of target genes at the next time point.^53^ To this end, various linear and non-linear regression models have been developed to infer GRNs. Dynamic GENIE3 (ref. 54) is the most popular tool developed using non-linear regression models based on random forest decision trees. This tool can handle time-series data to infer GRNs. OutPredict^55^ is another random-forest-based method that can incorporate priors (curated regulatory data) together with the dynamic time-series data. It has been successful in inferring causal edges between a TF and a target gene. OutPredict has been applied on *Arabidopsis* datasets and shown improved predictive accuracy compared to other state-of-the-art methods. Overall, generating a GRN is a useful way to explore the dynamics of TF-target interaction and explore regulation at different levels of expression. Nevertheless, it is always crucial to test the relevance of predictions biologically by comparing predicted interactions with the experimentally validated interactions. A well-refined GRN can be a key to biosynthetic pathway discovery.

### 3.5 Harnessing advanced dynamic regulatory networks for pathway discovery

Until now, biosynthetic pathway discovery in prokaryotes and ‘lower’ eukaryotes has been based on three principles, (i) identification of BGCs (ii) coexpression of genes, and (iii) coregulation, based on shared TFBS. In plants, however, gene regulation is characterized by highly promiscuous TFs.^56–58^ These TFs are also subjected to large-scale duplication and diversification, which make gene regulation in plants difficult to predict. It is therefore important to first resolve hidden gene interactions that facilitate pathway discovery. Transcriptional networks based on GRN models help scale down thousands of genes into small gene clusters. GRN models allow us to traverse through the gene regulatory hierarchy, in which multiple TFs regulate gene expression either directly, or indirectly by interacting first with other TFs and then with the target genes. This knowledge of TF-target gene interactions from GRNs may enable better prediction of co-regulated gene clusters and may improve the pathway discovery process by fine-tuning coexpression networks. To this end, it is also essential to include additional regulatory data like chromatin accessibility and small RNAs, which further help in refining the GRN model. Research on post-transcriptional control of gene expression by small RNAs has been gaining momentum with the advancement of sequencing technologies. So far, many published reports^59,60^ have established the impact of epigenetics on gene expression and regulation. Through the advancement of high-throughput sequencing technologies, various genome-wide assays have been developed to decode the epigenetic landscape of plants and examine chromatin accessibility.^61^ Seminal work on yeast and mammals has emphasized the importance of chromatin remodelers in the regulation of metabolic gene expression.^62^ In *Arabidopsis*, work by Yu *et al.* has shown that many biosynthetic gene clusters are characterized by unique chromatin signatures namely, histone 3 lysine trimethylation (H3K27me3) and histone 2 variant H2A.Z. These chromatin signatures are associated with the activation and repression of gene clusters in different plant tissues.^63^ This work has further demonstrated that knowledge of such chromatin signatures can be useful in mining plant genomes and identifying gene clusters that encode metabolic pathways. In addition to chromatin accessibility, 3D architecture of the genome may play a crucial role in localizing distantly coexpressed genes in proximity so that they can be co-regulated. The physical linkage of distant genes also allows TFs to co-localize as close as possible to their target genes to increase the transcriptional output with a limited TF protein concentration.^33^ Importantly, areas of open or active chromatin form loops or genomic compartments called Topologically Associated Domains (TADs), which range from tens to hundreds of kilobase pairs along the genome.^64^ This kind of compartmentalization affects the way genes are localized and regulated. Genes within a TAD show more similar expression patterns^65^ and it has also been demonstrated that long-range enhancer activities do not extend beyond a TAD. Interestingly, it has been shown that BGCs of some of the known metabolic pathways in *Arabidopsis* reside in local interactive 3D chromosomal domains, which show different topology in different tissues of the plant.^66^ Furthermore, comparative analysis of unrelated metabolic gene clusters revealed that TAD formation is a ubiquitous feature of the plant kingdom.^67^ Combining information on chromosomal organization and chromatin accessibility together with refined GRN models of pathway-encoding metabolic genes provides an unprecedented opportunity to mine plant genomes and elucidate biosynthetic pathways based on shared regulatory features.

## 4. Metabolomics

Metabolomics-based approaches such as chromatography coupled to mass spectrometry have long been applied to study specialized metabolites and explore biosynthetic pathways of interest in plants. With metabolomics, it is possible to identify pathway intermediates, and by applying multiple treatments, it is also possible to capture the spatiotemporal distribution of metabolites in different plant tissues. For example, with the initial characterization of diterpenoid phytoalexins such as momilactones (known to be allelopathic) in 2007, the complete momilactones pathway was reconstructed using *Nicotiana benthamiana* as a heterologous system.^68^ Various analytical and computational metabolomics approaches have been developed in the past decades to perform high-throughput profiling of specialized metabolites and to decode different biosynthetic pathways. However, despite a remarkable technological revolution in instrumentation, software, and databases, plant-based natural product discovery using metabolomics is still challenging for two main reasons. First, specialized metabolites show large functional and chemical diversity. Second, the proportion of specialized metabolites produced by a plant is a part of its total metabolome that can also consist of microbial metabolites, such as those produced by endophytes. It is estimated that, until now, only ∼6% of the total plant metabolic structural diversity has been cataloged in the Dictionary of Natural Product (DNP https://www.dnp.chemnetbase.com/faces/chemical/ChemicalSearch.xhtml).^69^ Here, we note that the recently introduced LOTUS (naturaL prOducT occUrrence databaSe)^70^ serves as a curated open-access alternative to chart plant-based chemistry amongst other natural products. LOTUS includes 700 000+ referenced structure–organism pairs which is twice the size of the DNP. It is surprising that despite such technological advancements, most of the plant-based chemical diversity remains elusive.

As the classical reductive approach (experimental and targeted approaches) to characterize metabolites is laborious and time-consuming, untargeted metabolomics using mass spectrometry (MS) has immense potential in performing wide-screen profiling of specialized metabolites and identifying an unprecedented number of metabolic classes from crude extracts.^71,72^ High-throughput identification of metabolites from multiple sources, for instance, leaf, root, soil, volatiles, *etc.,* have fueled the discovery of biosynthetic pathways by enabling the identification of key changes in the metabolite profiles. A full metabolite scan using chromatography coupled with MS (a.k.a. MS1 mode) has the advantage of (more accurately) quantifying metabolites but suffers from unreliable metabolite annotation as several compounds can have the same mass but a different molecular formula or may have the same molecular formula but differ in their chemical structures.^73^ To address this challenge, metabolites are further subjected to tandem MS mode (a.k.a. MS2 or MS/MS, or sometimes MS^n^ when deeper fragmentation levels are included) to generate a fragmentation spectrum of metabolites. Metabolites can be structurally annotated and readily identified using acquired MS2 fragmentation data using a plethora of software and tools that have been developed to mine and annotate such data.^74–76^

### 4.1 Current state of the art

Structural annotation of fragmentation relies on common substructures in different metabolites that share a common core biosynthetic pathway. Substructures here refer to a building block, functional group, or a scaffold within a chemical structure. Multiple software tools like MAGMa,^77^ MESSAR,^78^ MS2LDA,^79^ and CSI:FingerID^80^ can be used for substructure discovery and annotation. Furthermore, ClassyFire^81^ and NPClassifier^82^ classifications can be used jointly with MolNetEnhancer^83^ and CANOPUS^84^ to predict assignment of metabolite features to chemical compound classes like peptides, saccharides, flavonoids, *etc.*, which do have some common structural elements within that category. A notable early example applied an integrated *in silico* metabolomics workflow on plant metabolomics data and provided interesting insights into chemical differences between two clades of the cosmopolitan *Rhamnaceae* plant family.^85^ In metabolomics, common substructures or chemical moieties frequently yield similar spectral patterns; therefore, spectral similarities can also be exploited to group several spectra together to form networks of fragmented features, known as molecular network (MN) or mass spectral networks.^86,87^ Networking algorithms implemented in tools like GNPS molecular networking^86^ and Spec2Vec^88^ are some of the metrics that are starting to be used to group/cluster plant metabolite mass spectra together. MN-based approaches and several other annotation methods have been implemented in the Global Natural Product Social (GNPS) molecular networking platform.^86^ Metabolic pathways in general involve changes in chemical structures, known as bio-transformations, which result in distinct mass shifts in the fragmentation spectra.^89^ This holds true also for specialized metabolic pathways. The differences in the fragmentation spectra are also reflected in the molecular networks, in which metabolites are clustered together based on their mutual similarity.^90^ In such a cluster, one known metabolite peak can help in the annotation of its neighbors, facilitating the discovery of unknown metabolites. To this end, various *in silico* tools, like Network Annotation Propagation (NAP),^91^ MolNetEnhancer,^83^ SIRIUS,^92^ and NetID,^90^ have been developed that exploit network topology to annotate unknown metabolites coming from mass spectral networks. Finally, Pathway Activity Level Scoring (PALS) was developed to predict pathway activity levels either based on a set of curated (plant) metabolic pathways or based on grouped metabolites following *in silico* analyses such as those performed by MN^86^ or MS2LDA substructure discovery.^93^ The above-described advances in computational metabolomics workflows have enabled a much deeper understanding of metabolomics profiles by adding structural information to mass spectral data. The selection of metabolite features to focus on during integrative omics approaches is another challenge that in part can be solved by using relevant substructure and chemical class information inferred by *in silico* approaches, but also by appropriate experimental design, for example by including several separate tissues or time-based series.

Importantly, to comprehensively annotate metabolomics profiles with structures, lack of available relevant reference compounds and relevant reference mass spectral libraries pose severe limitations. For example, the reference compounds which are commercially available only cover a small spectrum of plant natural product diversity. Despite the recent advances in computational metabolomics and *in silico* metabolite annotation methods,^76^ complete structural identification of plant metabolites remains elusive. Notably, preparative-scale purification and *de novo* structural determination by nuclear magnetic resonance (NMR) spectroscopy are useful complementary approaches.^94–96^ However, collecting sufficient pure materials from complex matrices for structural elucidation is still challenging.

### 4.2 Time-based metabolomics

Monitoring the dynamic abundance of metabolites in plants during pathogen infections or crosstalk with the surrounding environments has attracted increasing interest in recent years. While most of the metabolomics-based biosynthetic pathway studies have focused on the traditional capturing of the metabolome data at a fixed-frame snapshot, time-based analysis has enormous potential to unveil transient metabolites both in terms of concentration and availability. A notable example to this end is the study^97^ conducted by Jeon *et al.*, where untargeted metabolomic data was generated for multiple treatments and the samples were collected at 12-, 24- and 48 hours post-infection to study the biosynthesis of falcarindiol in response to biotic elicitors; the data clearly indicated that several specialized metabolites, including falcarindiol, were only observed in specific combinations of time points and conditions. In another study,^98^ a time-based untargeted metabolomics experiment was set up to scan changes in metabolomic profiles during the germination of FGSC A4 conidiospores of the model fungus *Aspergillus nidulans*. Here, swelling in conidiospores was observed between 2 and 4 hours followed by the formation of a germination tube at 5 hours post-incubation. In the same study, cluster analysis of the metabolomic data demonstrated distinct clusters of samples taken at 2, 4 and 5 hours. Other clusters also clearly described phases of conidiospore germination. This pattern of clustering clearly showed that the time-series design discretely captured a switch in the metabolic abundance from swelling to germination and later developmental stages.

Statistical models can be used to infer the involvement of metabolite(s) in a biosynthetic pathway(s) based on such data. One limitation in this approach is the availability of time-points in metabolomics data due to inherent experimental costs or associated ethical considerations. According to Jendoubi *et al.*, for time-series-based metabolomics datasets, in general, less than ten time points are available. This is in contrast to the relatively large number of metabolomic variables available at each time point.^99^ The short experimental measurements hinder the model from fitting to new data and result in high generalization errors. Moreover, with such models, it is also difficult to find interesting patterns due to the non-collinearity of metabolites along the time points. To account for errors generated from multiple testing, MetaboClust^100^ was developed as an unsupervised clustering-based pipeline to handle time-based MS data.

**Fig. 4:**
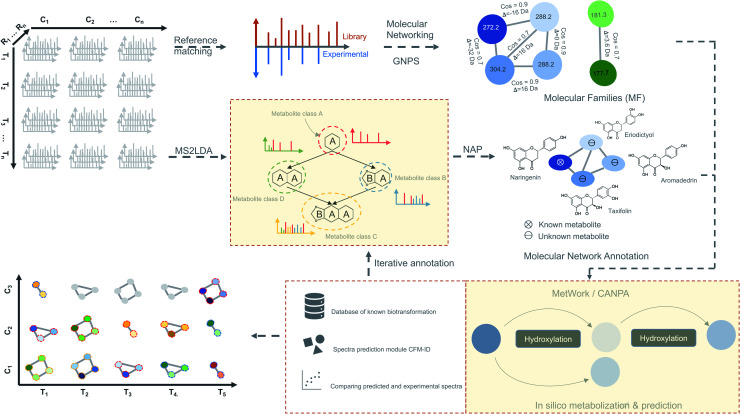
Time-based metabolomics data analysis. Molecular networks are generated using spectral data from all the MS^2^ samples by classical or feature-based molecular networking implemented in the GNPS.^86^ Additionally, spectral data are also subjected to the substructure discovery using MS2LDA^79^ and NAP.^91^ Metabolite annotation is further extended by *in silico*-metabolization method implemented in the MetWork^101^ pipeline. In addition, MetWork also proposes CFM-ID-predicted MS/MS spectra of the derivatized substrates. The time-series design is then applied to all the samples to check the distribution of differentially abundant metabolite (DAMs) across timepoints and across different conditions to better predict biosynthetic pathways.

We propose as a viable approach that instead of inferring biologically significant metabolites directly from the time-based statistical models, MF can be first generated from spectral data by considering individual time-point as an independent dataset (Fig. 4). Using this approach, a plethora of tools for metabolite identification and annotation are available to handle independent datasets. The annotation can be further enhanced by the availability of tools like MetWork/CANPA,^101^ which aids in metabolite annotation using *in silico* metabolization and prediction of bio-transformation reactions. Later, the annotated MFs can be mapped to the rows and columns of the time-series matrix (Fig. 4). By using a time-based statistical model, causal relationships between the time-points can be predicted. Such relationships, along with predicted bio-transformations, further facilitate the discovery of metabolic pathway(s) by uncovering the dynamic temporal patterns of the metabolites.

### 4.3 Pathway discovery using metabolomics

For pathway discovery, accurate identification, and annotation of metabolites as represented by mass features is a major challenge. This is due to the presence of isomers for many natural products which cannot be differentiated by their *m*/*z* values. This process can further be blurred by the existence of multiple empirical formulas for a single mass feature within a small *m*/*z* variance window (<5 ppm). Numerous public and propriety databases (discussed above) are available that store spectral and structural information which can be used to annotate metabolites by spectral matching (Fig. 4) of experimental to known compounds in the database.

A crucial step forward is the use of chemical ontology terms that are significantly enriched in metabolites and can be correlated with chemical classes. BiNChE^102^ has adopted ChEBI (Chemical Entities of Biological Interest) ontologies to link biological entities in the form of chemical classes to small molecules, facilitating the expansion of chemical space in pathway databases. In a similar way, ClassyFire converts traditional molecular descriptors such as SMILES (Simplified Molecular Input Line Entry System) or InchiKeys to well-structured hierarchical ontology terms. The incorporation of metabolite classification tools into annotation pipelines based on the principle of molecular networking facilitates the integration of structural information on metabolites. MolNetEnhancer was developed exactly with this goal in mind; it combines GNPS molecular networking tools with ClassyFire and other annotation algorithms in a single analysis pipeline. ChemRich is another interesting pathway-independent approach in this direction, which defines related molecules in modules using MeSH annotations and Tanimoto indexes.^103^ These modules are then subjected to a Kolmogorov–Smirnov (KS) enrichment test. Another possible route is to reconstruct plant metabolic networks first, after which pathways could be discovered using graph-based algorithms.^104^

All these advances in computational metabolomics have sped up metabolite identification, annotation, and pathway prediction. The biological complexity of plant systems still poses a great challenge to the pathway discovery from untargeted metabolomics data alone. It is therefore not surprising that integration of data from different omics-based platforms has shown great promise in achieving a better understanding of biological systems, specifically pathway discovery.

## 5. Integrative omics approaches for plant biosynthetic pathway discovery

Single-omic technologies like genomics, transcriptomics or metabolomics are adept at capturing fluctuations of individual components of specialized metabolism under specific conditions.^105^ Often, the measurement and spatiotemporal distribution of these components provide sufficient data to generate a focused set of hypotheses regarding the enzymes and/or metabolites involved in a biosynthetic pathway. A major challenge, however, remains the identification of biosynthetic pathways that comprise a complex network of reactions with metabolites as substrates and products and gene products as enzymes that catalyze the corresponding reactions. Single “omic” correlation-based approaches depend to a large extent on the availability of known genes and metabolites as a starting point. However, finding pathways for unknown genes and metabolites is not trivial and requires efficient integration of omics datasets to facilitate deeper system-level insights. In the past, multiple studies have adopted hypothesis-driven omics integration, in which some hypothesis was generated from a single omic dataset followed by validation using another omic dataset. For example, the biosynthetic pathways for podophyllotoxin in mayapple^22^ and 4-hydroxyindole-3-carbonyl nitrile in *Arabidopsis*^23^ were characterized by first identifying genes involved in the pathway of interest using transcriptomics-based approaches and later validated by targeted metabolomics. Such sequential approaches constitute an important step towards integrative omics analyses in which genomics, transcriptomics and metabolomics are combined to provide a holistic view of the system. Additionally, with the technological advances in gene and metabolite profiling, the application of combined omics technologies (integrative omics) has become less expensive. From Fig. 1, it is evident that many recent studies related to specialized metabolites biosynthesis have adopted an integrative strategy. The characterization of triterpenes is a classic example of how the technological revolution has paved the way for biosynthetic pathway discovery. Before the rise of coexpression-based genome mining analyses, thalianol was the only specialized metabolite known to be expressed in the roots of *Arabidopsis*. Later, three more pathways, namely those for marneral, tirucallanediol, and arabidiol, were characterized by combining genome mining, transcriptomics, and metabolomics, and recent analyses showed that additional triterpenes (arabidin, thalianin, and thalianyl fatty acid esters) are produced through the action of enzymes encoded elsewhere in the genome or by other gene clusters (through pathway crosstalk). The specialized metabolites from these biosynthetic pathways were shown to be functionally involved in the assembly and maintenance of *Arabidopsis*-specific root microbiota in response to pathogen infection.^106^

Below, we outline different types of omics integration strategies based on the design of the study and discuss the usage of these methods to improve the discovery of plant biosynthetic pathways.

### 5.1 Targeted pathway discovery

Here, to start with, a known gene encoding a biosynthetic enzyme is used as bait in downstream transcriptomic analysis to identify other coexpressed genes coding for enzymes that may be involved in the pathway based on the structural knowledge of the final metabolic product. Using this approach, Rajniak *et al.*, reconstituted the complete cyanogenic glucoside biosynthetic pathway of *Arabidopsis*.^23^ They started with a single pathogen-induced CYP450 (CYP82C2) gene and applied coexpression analysis and untargeted metabolomics to unveil a 4-hydroxyindole-3-carbonyl nitrile (4-OH-ICN) metabolite in *Arabidopsis*. Enzymes catalyzing intermediate steps in 4-OH-ICN biosynthesis were also identified using coexpression analysis, and their functions were validated using a heterologous expression system. A similar approach was adopted to uncover the complete biosynthetic pathway of etoposide aglycone,^22^ starting with 3 of the 4 previously characterized genes in *Podophyllum hexadrum* (mayapple). Similar to the known genes, known metabolites can be used as bait, as documented in the metabolomics-based pathway discovery section, for the targeted discovery of a pathway by annotation transfer or by spectra clustering. Although targeted approaches have been successful in the elucidation of pathways, dependency on a known gene/metabolite as starting point is a major limitation.^107^

### 5.2 Untargeted pathway discovery

Untargeted pathway discovery, also known as *de novo* pathway discovery, constitutes the prediction of novel pathway(s) from coexpression or metabolic networks. Using this approach, by mining transcriptome and metabolome of *Cameilla sinensis*, Li *et al.* observed that the expression of four genes—GOGAT, AIDA, GS and TS—was significantly correlated with the concentrations of ethylamine, glutamine and theanine. These genes and metabolites were shown to be involved in the theanine biosynthetic pathway and the correlations among them demonstrated the leaf metabolite variability in three colors and developmental stages.^108^ The untargeted analysis mainly made use of similarity networks of coexpressed genes and/or co-abundant metabolites, in which nodes represent genes and/or metabolites and the edges represent the strength of correlation. Work by Jeon *et al.* is arguably the most compelling example of an untargeted pathway discovery. They used a combination of time-based untargeted metabolomics and transcriptomics to reconstitute the biosynthetic pathway of falcarindiol production, which is a prototypical acetylenic lipid commonly found in carrot, tomato, celery and known to inhibit fungal development on plants and growth of human cancer cell lines.^97^

### 5.3 Methods of multi-omics integration and pathway discovery

#### 5.3.1 Unsupervised multi-omics integration

With the reduced cost of sequencing, more plant genomes are being sequenced and are becoming readily accessible to the scientific community. Such availability of genomic resources facilitates comprehensive mining of plant genomes for the identification of genes encoding novel pathways. However, the prediction of reactions catalyzed by the encoded enzymes, as well as of the corresponding pathways, represents a major hurdle. To this end, an unsupervised correlation-based method of omics integration would provide a potential solution that compares multiple features (gene, protein, or metabolite) and combines complementary information that comes from different omics-based platforms. Urbanczyk-Wochniak *et al.* reported the first instance of a pairwise transcript-metabolite correlation strategy where significant known and novel transcript-metabolite correlations were observed. However, depending on thresholds used, the correlation-based method was also prone to high false-positive or false-negative pairs, as correlations are often imperfect. Out of 26 616 correlated pairs, only 571 were found to be significant.^109^ The authors also observed pairing of a single transcript with multiple metabolites, for example, pairing of aminotransferases with both fructose-6-phosphate and glucose-6-phosphate, which are integral metabolites of sugar metabolism in potato tuber. Although such metabolite links can be hypothesized in many possible ways including novel links (like sharing a common cofactor or a TF-target gene interaction) between pathways, experimental validation is required to completely reconstruct the underlying correlations. Nevertheless, the approach of correlating transcript and metabolite data constitutes a potentially powerful tool in the discovery of biosynthetic pathways.

##### 5.3.1.2 Combination of single omics

Like transcriptomics and metabolomics, other combinations of single omic approaches have been successful in elucidating biosynthetic pathways. For example, despite the biochemical characterization using metabolomics,^110^ the genetic basis of the glaucousness in wheat and barley due to the deposition of wax on the surface of leaves, stem, and spikes remained elusive. Using genomics and transcriptomics, a metabolic gene cluster in wheat and barley that catalyzes β-diketones biosynthesis responsible for the biosynthesis of wax has been identified.^111^ In a similar way, a combination of genomics and metabolomics can also be used to localize genes responsible for a biosynthetic pathway. However, profiling a complete set of bona fide genes is generally not possible without transcriptomics data (Fig. 3B). To this end, a draft genome sequence of opium poppy chemotype Roxanne has been assembled recently to characterize BGCs spanning a 1-Mb region encoding the thebaine biosynthetic pathway.^112^ Essential steps in the thebaine pathway have been later confirmed using high-resolution metabolomics. Critically, the availability of the plant genomes in public repositories has allowed the direct investigation of biosynthetic pathways not only in the target species but also in phylogenetically closely related species. Availability of such genomic resources in the form of publicly available genomes has made it possible to effectively combine transcriptomics and metabolomics, making use of the genome annotations as reference data. In the last five years, 13/20 pathways (Fig. 1) were discovered using multi-omics based on transcriptomics and metabolomics. For instance, Zhan *et al.* have made use of the plethora of genomic resources available for rice and performed a metabolite-based genome-wide association study (mGWAS) to investigate the natural variation of 5,10-diketo-casbene biosynthesis in rice using *japonica* and *indica* subpopulations. Interestingly, a strong association was found between a diterpene gene cluster (five genes spanning 140 kb) on chromosome 7 (DGC7) and the japonica subpopulation. Further coexpression analysis revealed a massive increase in expression of the DGC7 genes upon being induced by methyl jasmonate, a potent inducer of defense responses in plants.^113^ The rationale for mGWAS is that the metabolite variants that are closely associated with genetic variants found in or close to the genes are likely members of the same pathway for the biosynthesis of specific metabolites. With this approach, novel pathways can be potentially identified without any prior information of a gene or metabolite. One limitation to this approach, which is related to GWAS, is the challenges involved in identifying candidate gene(s) within an often still large linked chromosomal region that are directly associated with metabolite variation. A potential solution is to perform further data mining to narrow down the list or look at public expression datasets to identify coexpression patterns of biosynthetic genes within the region. Rai *et al.* adopted a multi-omics approach to associate flavonoid glycosylation with plant stress response hormones. They combined profiles of transcripts and metabolites together with genomics-based promoter network analysis of DEGs of *tt8* mutant lines. This combined analysis identified links between the biosynthetic pathways of specialized metabolism in *Arabidopsis*.^3^ Similarly, Toghe *et al.*, characterized a flavonol-phenylacyl transferase 2 (FPT2) enzyme using genome sequence analysis with transcript and metabolite profiling. This enzyme catalyzes an important step in saiginol biosynthesis and provides tolerance against UV light.^114^

Crucially, despite its effectiveness, the integration of paired-omics data is challenging for multiple reasons. First, it is difficult to link every transcript with a metabolite as a time-lag exists between gene expression and metabolite availability. Second, the rate of false-positive mappings, using pairwise correlation methods as described above, can be very high. Third, and most importantly, an effective experiment design is very crucial to decrease the occurrence of false-positive pairings while combining transcriptomics and metabolomics data. Cavill *et al.*, have argued that the best experimental design for omics integration is the one where the original sample is divided into two batches (split-sample approach) as compared to repeated, replicate-matched and source-matched designs. In the split-sample approach, after the split, the first batch is used to generate transcriptomic data, and another batch is used to generate metabolomic data.^115^ Such paired- or linked-omics data sets account for the biases originating from sample replication^116^ and reduce false-positive rates. The study by Jeon *et al.* on falcarindiol biosynthesis in tomato, discussed in more detail earlier in this review, provided a notable example of a time-based linked transcriptomics and metabolomics study. This approach facilitated the discovery of biosynthetic pathway enzymes without prior knowledge of any genes within the pathway studied. This suggests that metabolite–transcript correlation analysis has the potential to identify candidate pathway genes and provides a solid foundation for characterizing biosynthetic pathways, provided the study setup was done in a systematic manner considering the above challenges.

##### 5.3.1.3 Other unsupervised methods

Other unsupervised methods, like factor analysis^117^ and clustering-based approaches,^118,119^ focus on the principal sources of shared variation in the omics data which can link multiple heterogeneous datasets like genomics, transcriptomics and metabolomics. Such methods have been successfully applied to study the environmental effects on grape berry composition^120^ and to uncover novel secondary metabolic pathway regulators in grapevine.^121^ Other multivariate unsupervised methods which are routinely used in multi-omics integration include Independent Component Analysis (ICA)^122^ and Canonical Correlation Analysis (CCA).^123–125^ The focus of unsupervised methods is to reduce the dimensionality of the data by finding a new (set of) variables using a linear combination of original variables. The variables here represent columns of the data matrix and rows are the individual observations. The new variables are also known as latent variables or components. These unsupervised methods can be applied to single and multiple omics datasets. For example, Liu *et al.* have applied ICA to human breast cancer proteomic and transcriptomic data and identified significant components (clusters) of meta-gene and meta-protein which can be associated with clinical features. A large portion of these associations between a signature component and a clinical feature indicated pathway-level information about the molecular mechanism underlying clinical features.^126^ Similar approaches can be easily adapted to uncover biosynthetic pathways in plants. Crucially, it is evident that unsupervised multi-omics data integration has some disadvantages and limitations as well. One common limitation is the high background noise that often conceals bona fide gene–metabolite associations. Importantly, most biosynthetic pathways are activated only under specific conditions; therefore, the abundance of specialized metabolites is very scarce, and it is challenging to capture them repeatedly even with time-based analyses. For this reason, more advanced omics data feature selection methods are required to aid unsupervised methods in enhancing the identification of gene-metabolite links with high precision and fine resolution.

#### 5.3.2 Supervised multi-omics integration (integrative omics)

Supervised multi-omics integration methods, unlike unsupervised methods, make use of the phenotypic labels of the sample, for example, methyl-jasmonate-treated and untreated samples or pathogen-infected and uninfected samples. Supervised methods then use a machine learning (ML) algorithm to train a multivariate model on integrated data to make predictions and classify the samples in different label categories. A gene or a metabolite membership to a pathway can be an example of a label for pathway discovery where predictions can be made for other genes or metabolites based on the labeled gene. This dependency on labels is a big challenge as only a few plant species have experimentally validated pathway annotations. Even in a well-labeled system, pathway representation of genes or metabolites is very scarce, and to train a supervised model you potentially need dozens of examples as a training dataset. To improve the overall accuracy of the prediction it is important to integrate data and select features which are most informative. To this end, Rohart *et al.*, have developed mixOmics to integrate several heterogeneous data from different platforms at once by applying different multivariate methods.^127^ These methods extend *Projection to Latent Structure* (PLS) models for discriminant analysis which can be used for the identification of molecular signatures. Although most of the studies used mixOmics on human systems, the methods in the package could be easily repurposed to plant systems.

Unlike other organisms, model-based multi-omics data integration has been scantly applied in plants due to their metabolic diversity, complex signaling networks, and poorly annotated large genomes. A notable example of supervised data integration in *Arabidopsis* is the work done by Zander *et al.*,^36^ where a time-based transcriptome and (phospho)proteome data were integrated to create a GRN using the Regression Tree Pipeline for Spatial, Temporal, and Replicate (RTP-STAR) data.^128^ Based on the GRN predictions it was established that jasmonate signaling shows crosstalk with many other signaling pathways, as 30–50% of genes from other hormone signaling were found to be targeted by MYC2, a master transcription factor in jasmonate signaling. Interestingly, De Clercq *et al.*^129^ have also generated an integrated GRN (iGRN) using a supervised learning approach, whose predictive performance outperformed state-of-the-art experimental methods in terms of recovering functional interactions. The iGRN correctly predicted new functions for hundreds of unknown TFs, including 13 novel regulators of the reactive oxygen species (ROS) stress response.

These examples suggest the possibility that multi-omics data can be integrated with ML to train pathway membership prediction models, at least for larger and more complex pathways. This can be done effectively either by direct feature integration or by ensemble integration. In the case of direct feature integration multiple features from different omics datasets, for instance transcriptomics and metabolomics, are concatenated in a single feature matrix to train pathway prediction models. In ensemble integration, features derived from single omics datasets are first used to build an omics-type-specific model. A prediction score is obtained for each feature using such a model. All prediction scores are later used as features to build a final pathway prediction model. This strategy has already been used to predict cancer types by integrating mRNA, miRNA and methylation data.^130^ In the context of plant pathway discovery, the multifactorial nature of the learning perhaps makes this method most suitable for complex and network-like branched pathways, in which enzyme members cannot be identified based on simple correlations.

### 5.4 Pathway-based integration

Another useful approach, especially in biosynthetic pathway discovery, is data integration using databases of known pathways. In this category, data integration can be performed using unsupervised or supervised methods. The crucial step, however, is to map information from the omics data onto the biological pathway repositories or reaction databases. The mapping is useful as it adds another layer of information which alleviates, to some extent, the false-positive association between multi-omics features. The biological databases covered in the metabolomics section of this review provide key information for pathway annotation and are the building blocks of software for multi-omics integration at the pathway level. For example, tools like MapMan and PathVisio have been developed to investigate and integrate multi-omics datasets from plants. MapMan^131^ has been applied to integrate transcriptomic, proteomic, and metabolomic data. This enabled successful mapping of omics data onto 123 out of 127 available KEGG pathways, and pathways such as the citrate cycle were shown to be highly enriched in this study. Similarly, PathVisio^132^ was used to study signaling pathways in *Arabidopsis*. They revealed that *Arabidopsis* mutants with high levels of methylerythritol cyclodiphosphate induce stress-response signaling pathways which include biosynthesis of jasmonate and salicylate. These examples show the applicability of pathway-based tools for elucidating the inherent modulation of certain biochemical pathways, especially in multi-omics studies.

## 6. Perspective and future directions

In the past few decades and since the discovery of the first biosynthetic pathways and gene clusters in plants, there has been a rapid increase in the discovery of plant biosynthetic pathways, which can be attributed to the massive technological advancements in sequencing and mass spectrometry technologies. However, compared to other biological systems like bacteria and fungi, the expansion of biosynthetic pathway discovery in plants is still lagging because of the functional diversity and structural complexity of biosynthetic pathways in the plant kingdom. Work done by Hickman *et al.*,^35,37^ Zander *et al.*^36^ and Huang *et al.*^106^ have reported intensive crosstalk between signaling pathways in *Arabidopsis* in response to certain abiotic and biotic environmental cues. This highlights a huge dynamic network of genes that work synergistically within one or more biosynthetic pathways. Additionally, the dynamics of TF-target interactions have also comprehensively changed our understanding of GRNs underlying plant responses. We propose that to disentangle such complex networks and dynamic events, efficient time-based experimental designs should be adopted for omics data generation, which account for temporal aspects of differential expression of genes and differential abundance of metabolites between time points and multiple conditions. For integrative omics, inclusion of time points is an effective methodology, as it ensures capturing linear and non-linear relationships between features, *e.g.*, gene and metabolites, from different omics datasets. The distinction between time points, gene expression, and metabolite abundance can explain how different genes and metabolites interact, and fill gaps in the understanding of substrates, products, and the enzymes that catalyzing the associated reactions. This aids in unwiring complex specialized metabolic pathways in plant systems.

Genomic architecture, in the context of biosynthetic pathway discovery, sheds light on the organization of biosynthetic genes within a genome. As discussed above, it is believed that more distal genes in plant genomes, involved in a biosynthetic pathway, could be brought together in proximity by chromatin folding, using TADs or chromatin loops, so that they can be coregulated.^66^ Along with the discovery of chromatin marks associated with long-range interactions, it has been proven that chromatin folding is a dynamic event;^133^ Therefore, we expect that the analysis of chromosomal organization will become more crucial in the context of discovering plant BGCs and the integration of genomic-based regulatory data like ChIP-seq/DAP-seq, ATAC-seq, and Hi-C with other omics discussed above will be useful in the discovery of biosynthetic pathways.

**Fig. 5:**
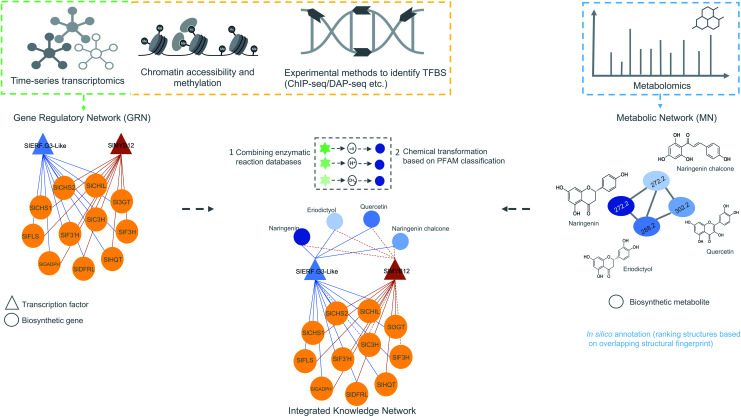
Overview of data integration possibilities to predict biosynthetic pathways. The figure is inspired on the MicroTom^135^ metabolic network where coexpression networks are correlated with metabolites to generate a knowledge network of flavonoid biosynthesis genes. Other genomic data like ChIP- or DAP-seq data can also be integrated with the transcriptomic data to obtain a holistic view of gene regulation of a biosynthetic pathways. Later, metabolomics data can be added to generate an integrated knowledge network (IKN). Reaction databases can be mapped to the IKN to predict biosynthetic pathways.

For efficient pathway discovery, effective data integration methods are needed that ensure significant association between features from multiple different omics data together in a biologically meaningful way. As supervised methods are more complicated and require phenotypic labels, which are poorly available for many non-model plant species, correlation-based data integration holds great promise in biosynthetic pathway discovery. Most recent studies have adopted correlation-based methods to integrate omics data because of its straightforwardness and ease of use. Recent studies on maize antibiotic biosynthesis^134^ and the development of the MicroTom Metabolic Network (MMN)^135^ are some of the notable examples where correlation-based integration has been successfully applied to integrate transcriptome and metabolome data (Fig. 5). False-positive rates from such approaches can be mitigated by implementing time-based experimental designs and prioritizing candidates based on regulatory information embedded within GRNs. Currently, no systematic, unsupervised multi-omics method has been developed that integrates genomic, transcriptomic, and metabolomic data for untargeted (plant) specialized metabolic pathway discovery. We anticipate that correlation-based and pathway-based data integration methodologies will soon be combined to predict biosynthetic pathways by correlating the expression of enzyme-coding genes with abundant metabolites sharing the time points or conditions in linked/paired transcriptomic–metabolomic experiments. To this end, it will be essential to also integrate a database of enzymatic reactions with annotations in the form of protein domains and associated catalytic enzymes for each reaction. The main impetus for such database integration comes from the need to scan whether the observed metabolic changes or biochemical transformations (which can be observed as mass shifts in metabolome data) can be explained by any experimentally validated reaction in the database that is linked to a catalytic enzyme and encoded by a gene present in a coexpression module. Reaction databases like RetroRules^136^ and Allchemy,^137^ are some of the notable examples that greatly emphasize the interest of using reaction rules from the known reactions not only in building metabolic models but also in predicting biosynthetic pathways. It is important to note here that, unlike RetroRules, Allchemy is proprietary database of reaction rules and therefore cannot be directly included in open-source pipelines. It is important to note that, unlike Allchemy, RetroRules is an open-source database of reaction rules and can be readily used and included in other open-source pipelines.

A limitation that may hinder effective data integration is cell-type specificity. Genome-wide studies generating omics data generally pool data from different cell types within the same tissue. This sort of sampling adds additional noise to the data, as it is well known that different cell types in plants have different transcriptional and metabolic dynamics, and each cell type responds uniquely to different environmental cues.^138^ Single-cell analysis both in transcriptomics^139^ and metabolomics^69^ offers great opportunities to explore cell-type specificity in terms of characterizing biosynthetic pathways. Alvarez *et al.*^33^ have also proposed the development of ML algorithms that can incorporate spatial and temporal information, in the form of an individual cell or tissue-specific omics data, to better predict regulatory interactions in GRNs^140^ which ensures improved pathway prediction. It is well known that accumulation of specialized metabolites, for instance defense-related compounds, in specific plant tissues or cell types, is a way to avoid autotoxicity reactions in the surrounding tissues. It also enhances the effectiveness of the compounds against attackers that function in a spatially specific manner.^141,142^ Therefore, it can be very beneficial to investigate the metabolic specialization in plants at the tissue or single-cell level to understand the spatial–temporal coordination of cellular processes underlying specialized metabolic pathways. A notable example in this direction is the work done by Li *et al.* (2016), where metabolomics and transcriptomics data were generated from 14 plant tissues and developmental stages of *Nicotiana attenuata* and predictions were made about the assignment of unknown genes and metabolites to specific metabolic pathways using the principles of information theory.^143^ The authors further validated the predicted function of two UDP-glycosyltransferases in flavonoid metabolism by virus-induced gene silencing.^143^ Very recently, Hong *et al.* (2022) have used paired-tissue-specific transcriptomics and metabolomics to elucidate the complete biosynthetic pathway of strychnine, a complex monoterpene indole alkaloid (MIA) from poison nuts (*Strychnos nuxvomica*).^144^ Additionally, in another recent study by Li *et al.* (2022), complete biosynthetic steps of another MIA have been identified in *Catharanthus roseus*.^145^ In this study, the *C. roseus* genome was first comprehensively improved using state-of-the-art sequencing technologies like Oxford Nanopore (ONT) and proximity-by-ligation Hi-C sequencing to find new clusters of biosynthetic genes involved in the MIA biosynthesis. Interestingly, 3D interactions between biosynthetic loci were also revealed using long-range chromosome interaction maps. Such differential non-random organization of gene clusters in 3D space contributes to coordinated gene expression in different plant tissues and cell types. Additionally, combining single-cell metabolomics and transcriptomics, a novel intracellular transporter, known as a MATE transporter, was identified that transports secologanin from the cytosol into the vacuole. This transport process is an important step responsible for the tissue specificity of the MIA.^145^ In addition, a reductase that was not previously known and responsible for the formation of an important intermediate in MIA biosynthesis, anhydrovinblastine, was identified.^145^ With these findings, this study demonstrated the effectiveness of single-cell multi-omics in biosynthetic pathway discovery.

Taking together all the advances and challenges we highlighted here, integrative omics approaches based on well-designed multi-omics experiments, with improved throughput and precision in detecting gene-metabolite associations, together with the availability of high-quality (well-annotated) plant (pan)genomes, will likely yield unprecedented opportunities for plant-based specialized metabolism research in the coming years.

## 7. Authors contributions

M. H. M., S. C. M. v. W., and K. S. S. conceived the review. K. S. S. wrote the manuscript. M. H. M., S. C. M. v. W., and J. J. J. v. d. H. supervised the writing of the manuscript and provided edits and suggestions for the improvement on all sections and figures. All authors proofread the entire manuscript.

## 8. Conflicts of interest

J. J. J. v. d. H. is a member of the Scientific Advisory Board of NAICONS Srl., Milano, Italy. M. H. M. is a member of the Scientific Advisory Board of Hexagon Bio and co-founder of Design Pharmaceuticals.

## Supplementary Material
